# Raccoon (*Procyon lotor*) biomarker and rabies antibody response to varying oral rabies vaccine bait densities in northwestern Pennsylvania

**DOI:** 10.1016/j.heliyon.2018.e00754

**Published:** 2018-09-06

**Authors:** Kerri Pedersen, Brandon S. Schmit, Thomas J. DeLiberto, Jason R. Suckow, Amy J. Davis, Dennis Slate, Richard B. Chipman, Robert L. Hale, Amy T. Gilbert

**Affiliations:** a U.S. Department of Agriculture, Animal and Plant Health Inspection Service, Wildlife Services, National Wildlife Research Center, 4101 LaPorte Avenue, Fort Collins, CO 80521, USA; b U.S. Department of Agriculture, Animal and Plant Health Inspection Service, Wildlife Services, 2150 Centre Avenue, Building B, Fort Collins, CO 80526, USA; c U.S. Department of Agriculture, Animal and Plant Health Inspection Service, Wildlife Services, National Rabies Management Program, 59 Chenell Drive, Suite 2, Concord, New Hampshire 03301, USA; d U.S. Department of Agriculture, Animal and Plant Health Inspection Service, Wildlife Services, 1777 Stagecoach Court, Powell, Ohio 43065, USA

**Keywords:** Ecology, Virology, Zoology, Environmental science

## Abstract

Distribution of oral rabies vaccine baits has been used as a strategy for managing rabies in the United States since the 1990s. Since that time, efforts have been made to improve baiting strategies with a focus on bait density to maximize both efficiency and cost effectiveness. An optimal rabies management strategy includes a vaccine bait preferred by the target species that is distributed at the minimal density needed to achieve population immunity to prevent rabies spread. The purpose of our pilot study was to examine the effect of 75, 150, and 300 baits/km^2^ vaccine bait densities on rabies virus neutralizing antibody (RVNA) seroprevalence in raccoons (*Procyon lotor*). Raboral V-RG® fishmeal polymer baits (Merial Inc. (now a part of Boehringer Ingelheim), Athens, Georgia) contain a tetracycline biomarker that was used to estimate bait consumption as another measure of intervention impact. Our results suggest that raccoon RVNA response increases as bait density increases, but the effect may not be sufficient to justify the cost except in the case of contingency actions or an epizootic. Non-target species, especially opossums (*Didelphis virginianus*) in certain areas, should be considered when determining an appropriate bait density to ensure sufficient baits are available for consumption by the target species.

## 1 Introduction

Prior to 1960, the majority of rabies cases in the United States (U.S.) were associated with domestic dogs [1], but in 2007 the U.S. was declared free of canine rabies virus (RABV) [2, 3]. Since then, wildlife have accounted for the majority, exceeding 90% of rabid terrestrial animal cases reported in the U.S. since the early 1990s [4,5]. Raccoons (*Procyon lotor*) are one of the primary terrestrial reservoir species for RABV in the U.S., and because they are ubiquitous, a spatially continuous susceptible raccoon population exists to allow westward movement of the virus [6, 7]. Raccoon RABV is enzootic throughout the eastern U.S., and expansion of this variant into the mid-Atlantic and northeastern U.S. has been attributed to a restocking program in the 1970s that resulted in transportation of rabid raccoons from southern states [8, 9, 10]. In the late 1970s, the raccoon RABV variant was discovered in raccoons on the border between West Virginia and Virginia, and subsequently spread throughout raccoon populations in northern Virginia, Maryland, and Pennsylvania. In Pennsylvania, the first rabid raccoon was documented in Bedford county in the south central region of the state in 1982 [8]. By 1996, raccoon RABV was enzootic throughout all 67 Pennsylvania counties.

Although the Appalachian Mountains may have served as a natural geographic barrier and deterred westward movement of raccoon RABV, oral rabies vaccination (ORV) zones were established to better ensure that raccoon RABV would not expand its range to the west [11]. Movement of raccoon RABV to naïve areas west of its current distribution could result in a rabies epizootic that would likely lead to increased mortality in domestic and wild animals, with increased risk of human exposure [12, 13].

Coordinated ORV campaigns have been used as a tool for managing RABV in the U.S. since the U.S. Department of Agriculture's National Rabies Management Program (NRMP) was established in 1997 [2]. The ORV program is an important strategy not only for preventing raccoon RABV from spreading to new, uninfected populations [14], but also in limiting the current distribution and ultimately eliminating this variant in the U.S. [15]. While the ultimate goal of ORV is reduction of cases and elimination of the raccoon variant, monitoring rabies virus neutralizing antibody (RVNA) response in target species prior to and after baiting, with a higher serologic response expected after baiting [16], remains a critical metric of program monitoring. Ideally, baits should be distributed at a density that will result in immunizing greater than 60% of the population against rabies [17, 18].

The objective of our pilot study was to compare the effect of aerially distributing oral rabies vaccine baits at target densities of 75, 150, and 300 baits/km^2^ in an ORV-naïve area on bait consumption and RVNA seroconversion rates in raccoons in three study areas of similar habitat types in northwestern Pennsylvania. Bait consumption was determined by examining the difference in tetracycline biomarker deposition in premolars and RVNA seroconversion rates of raccoons estimated before and after ORV.

## 2 Materials and methods

### 2.1 Ethical approval

Approval for conducting the experiments in this study (QA-983) was obtained from the U.S. Department of Agriculture, Wildlife Services, National Wildlife Research Center's Institutional Animal Care and Use Committee on 15 July 2002.

### 2.2 Study area

**Fig. 1:**
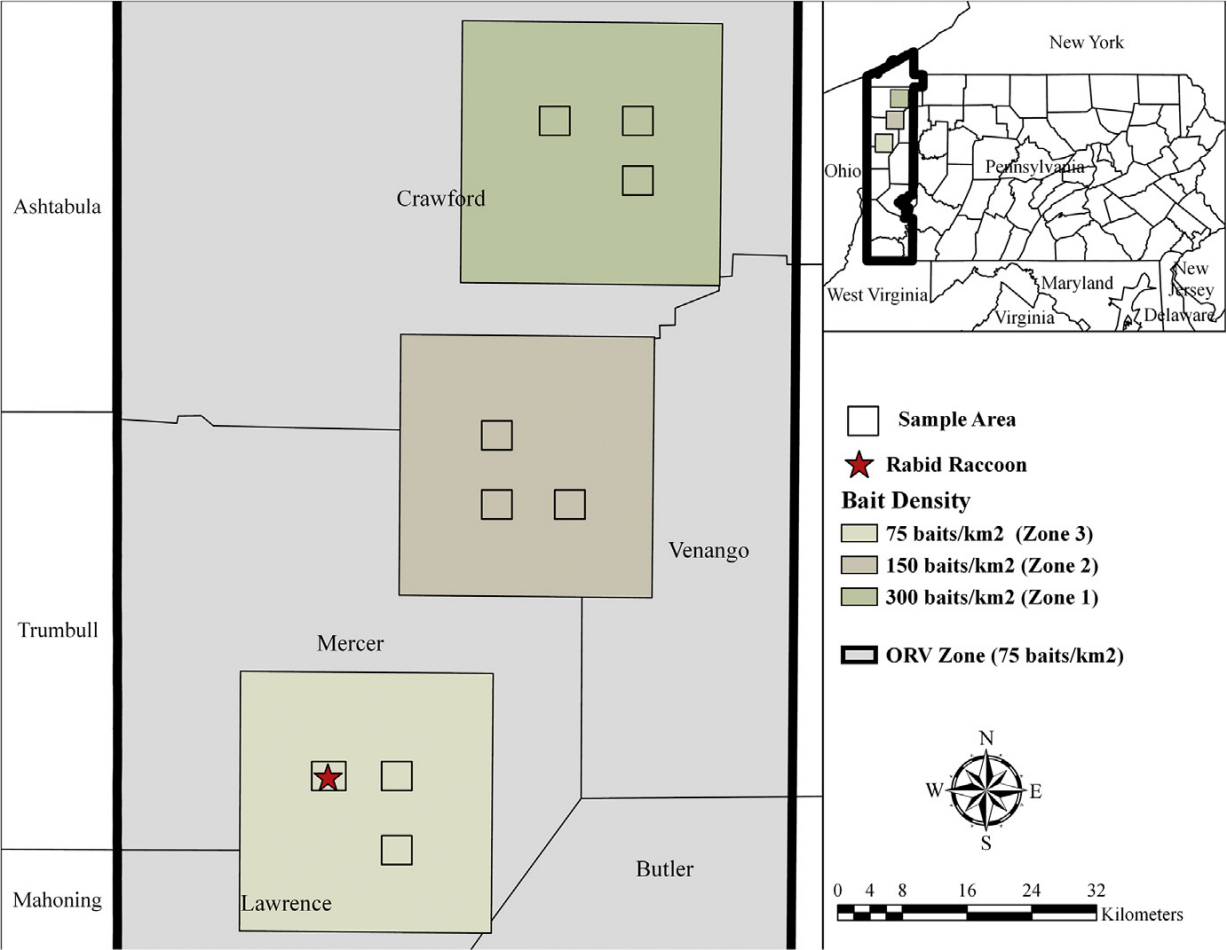
Location of three study zones in Pennsylvania where oral rabies vaccine (ORV) bait densities of 75, 150, and 300 baits/km^2^ were distributed in 2002. The three zones are located within a larger area that was treated with ORV at 75 baits/km^2^.

The study area was located in Mercer and Crawford counties in northwestern Pennsylvania and consisted of three 583 km^2^ zones of similar habitat types (primarily deciduous forest, pasture, and cultivated crops (as determined by the National Land Cover Database [19])), and where baits had not been distributed previously (Fig. 1). Although baits were distributed in Ohio and Pennsylvania in 2001, our study zones were placed a minimum of 3.5 km from the outer edge of where ORV baiting had occurred previously. In the center of each ORV bait density zone, a 65 km^2^ study area was delineated that included three 2.6 km^2^ sampling areas. Study areas were located ≥4.8 km apart with an 8 km buffer to the edge of the zone to ensure raccoons trapped within each study area were exposed to the appropriate bait density (Fig. 1). Two zones in Mercer County were baited at a density of 75 and 150 baits/km^2^, respectively. The third zone was located in Crawford County and baited at a density of 300 baits/km^2^.

### 2.3 Bait and bait distribution

The vaccinia-rabies glycoprotein recombinant vaccine (Raboral V-RG®; 1.5 mL; Merial Inc. (now a part of Boehringer Ingelheim), Athens, Georgia) was encased in a plastic sachet inserted into a hollow cube of fishmeal polymer bait, and sealed with paraffin (Bait-Tek, Inc. Orange, Texas, USA) [20]. Tetracycline (150 mg) was incorporated into the bait matrix to serve as a biomarker for determining bait consumption, which is detectable in the cementum of the teeth two days post-consumption [21].

Baits were distributed with fixed-wing aircraft from 16–18 August 2002, at approximately 150 m above ground level at 145 knots with 500 m spacing between parallel flight lines. Baits were distributed at target densities of 75, 150, or 300 baits/km^2^ in the corresponding study areas, and at 75 baits/km^2^ across the surrounding 16,978 km^2^ ORV zone (Fig. 1).

### 2.4 Animal trapping and sampling

To measure intervention impact as a function of bait density, pre-bait trapping was conducted to determine baseline levels of tetracycline biomarker and RVNA in raccoons within study areas. Post-bait trapping occurred approximately four weeks after ORV distribution, which has been demonstrated as sufficient time to develop an antibody response [22].

Safeguard live traps (Safeguard Products, New Holland, Pennsylvania, USA) were baited with marshmallows coated with anise oil. Five traps were placed at 268 m intervals along each of five transects of 322 m width for a total of 25 traps per study area. Traps were checked daily for 10 and 14 consecutive days during pre-bait and post-bait capture intervals, respectively.

From 5–25 July 2002 and 16–30 September 2002, 289 unique raccoons were sampled. Five raccoons were captured and sampled in July and September. Live-trapped raccoons were anesthetized with a 5:1 mixture of ketamine (10 mg/kg) and xylazine (2 mg/kg) injected intramuscularly [23]. Each raccoon was tagged with a unique metal ear tag (National Band and Tag Company, Newport, Kentucky) for identification, and sex, age, weight, and capture location were recorded for each animal. A blood sample was collected from a peripheral vein, and the first premolar tooth was collected from a subset of anesthetized animals. In July, during pre-bait sample collection, raccoons were released at the point of capture unless serious injuries or signs of abnormal behavior were observed. Samples were not collected from raccoons that were recaptured during July or after they had been sampled once in September. Injured or abnormally-acting animals were humanely euthanized under heavy anesthesia in accordance with the American Veterinary Medical Association's Guidelines on Euthanasia with an overdose of pentobarbital (Beuthanasia-D Special, Merck Animal Health, Madison, NJ) or potassium chloride injection [24]. In September during post-bait trapping, all captured raccoons were anesthetized and then euthanized following sample collection and the head was subsequently removed for rabies testing (n = 154). Non-target species were released at the point of capture without chemical immobilization or sampling.

### 2.5 Rabies diagnostics

Blood was centrifuged on the same day of collection and serum was separated and submitted to the Centers for Disease Control and Prevention in Atlanta, Georgia for screening. Samples were tested for the presence of rabies virus neutralizing antibodies (RVNA) using the rapid fluorescent focus inhibition test (RFFIT) [25]. Sera were screened for RVNA at dilutions of 1:5 and 1:25. Raccoons with a titer >5 (≈0.05 international unit per milliliter (IU/mL)) were considered antibody positive, representing >50% reduction of fluorescing foci at a 1:5 serum dilution [26]. Due to the delay in reporting our results, the positive control titers necessary to convert the serology results to IU/mL were no longer available.

The entire raccoon head was submitted to the Pennsylvania Department of Agriculture Veterinary Laboratory in Harrisburg, Pennsylvania where the presence of RABV antigens in brain tissue was determined using the direct fluorescent antibody test [27].

### 2.6 Aging and biomarker analysis

Premolar teeth were submitted to Matson's Laboratory LLC (Manhattan, Montana, USA) for age determination and tetracycline analysis. Age was determined using a compound microscope and ultraviolet (UV) light with filters to detect cementum or dentin annuli [28]. Raccoons less than one year were classified as juveniles, and those one year or older as adults. The premolars were cut longitudinally to obtain a thin section for biomarker analysis using a specially prepared compound microscope. An ultraviolet filter was used to detect fluorescence in the premolars created by the tetracycline biomarker [28]. The proportion of raccoons with biomarker was used as a measure of ORV bait exposure and consumption before and after bait distribution, respectively.

### 2.7 Data analysis

**Fig. 2:**
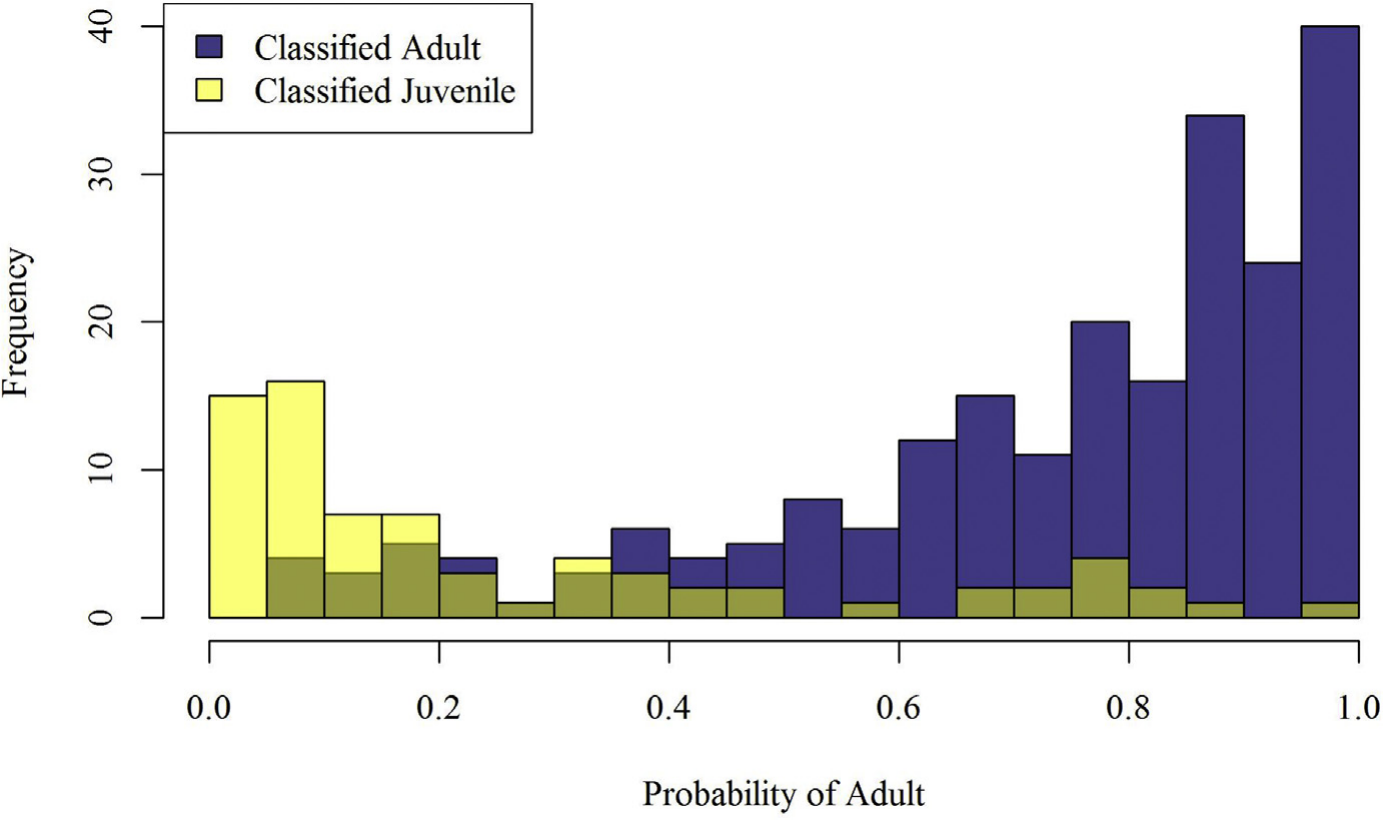
Histograms of raccoons (*Procyon lotor*) sampled in Pennsylvania and assigned a probability of being an adult based on sex, weight, and time of year captured. Raccoons classified in the field as adults (blue bars; n = 191) are shown, along with raccoons classified in the field as juvenile or unknown (yellow bars; n = 73). The light green is where the two histograms overlap. In the analysis, raccoons with a less than 25% probability of being an adult were classified as juveniles, raccoons with a greater than 75% probability of being an adult were classified as adults, and raccoons with a 25–75% probability of being adult were categorized as unknown.

Due to apparent anomalies in the age classifications recorded at the time of capture, a secondary method was used to estimate age. Age estimates based on cementum annuli counts were available for 30 raccoons. For the remainder of the samples (n = 264), a data set of raccoons (n = 1,584) collected in Virginia during approximately the same months as this study but over three sampling years (2014–2016; unpublished data) were used to develop a relationship between ordinal date, sex (0 for males, 1 for females), and weight that distinguished adults and juveniles well (AUC statistic = 0.81). Using this equation, (logit(probabilityofbeinganadult)=−1.7+1.3∗weight+1.3∗sex−0.02∗date), we estimated the probability of raccoons being an adult in our study (Table 1). The probabilities were then plotted in separate overlapping histograms by the original age classification (Fig. 2). To allow for more uncertainty than using a strict cutoff value and to account for any latitudinal variation in weights between Virginia and Pennsylvania, we assumed all raccoons with a less than 25% probability of being adults were juveniles, and all raccoons with a greater than 75% probability of being adult were adults, and then categorized all individuals where the probability of being adults from 25-75% as unknown. Since the impact of age effect might be influenced based on our selection of a cutoff value for age classification, we looked at the sensitivity of the cutoff on the age effect in the model selection. None of the age classifications we examined were competitive models for the difference in RVNA seroprevalence, unless we used the original age classifications recorded at the time of capture.

**Table 1:** Beta estimates, standard errors (Std error) and test statistics for the covariates that relate to the probability (Pr) of a raccoon (*Procyon lotor*) being an adult based on 1,584 raccoons sampled in Virginia from 2014–2016.

Co-variates	Estimate	Std error	Z value	Pr(>|Z|)
Intercept	−1.6593	0.3808	−4.36	0.0000
Weight	1.2581	0.0788	15.97	0.0000
Sex	1.2587	0.1392	9.04	0.0000
Day of year	−0.0193	0.0015	−13.14	0.0000

Although we had few raccoon recaptures between trapping periods, we wanted to estimate raccoon densities to calculate the number of baits available for consumption. We used closed population capture-recapture models implemented in Program MARK which assumed a population closed to births, deaths, emigration, and immigration to estimate raccoon densities by zone [29, 30]. A behavioral effect on capture rates (e.g., trap happiness or trap shyness) was included by estimating initial capture rate and recapture rate separately and allowing for population size to vary by study zone. Abundance was converted to density by dividing by the study area (7.8 km^2^), and variance was converted using the Delta method [31].

A simple linear model was used to examine the difference in RVNA seroprevalence and tetracycline deposition in the teeth between pre-bait and post-bait trapping. We evaluated the impacts of average difference in age, sex, and weight on antibody response. Additionally, we examined several hypotheses about the relationship of bait density and change in RVNA occurrence including a positive linear relationship. One hypothesis was that there would be a difference between 75 baits/km^2^ and 150 baits/km^2^, but little difference between 150 baits/km^2^ and 300 baits/km^2^ (Den75). Another hypothesis was that there would be little difference between 75 and 150 baits/km^2^, but there would be a difference between 150 and 300 baits/km^2^ (Den300). We also considered that there would be a difference between all levels of bait densities but the difference between 75 and 150 baits/km^2^ would not be the same as that from 150 to 300 baits/km^2^ (Denfac). Model selection was performed using Akaike's Information Criterion (AICc) and correcting for small sample sizes [32]. There was little support for any of the additive models examined (all model weights for additive models were <0.05); therefore, we present the results for the top ten models only (all with a model weight of ≥0.01). Models were fit and implemented in R (R Project for Statistical Computing, Vienna, Austria) [33].

## 3 Results

### 3.1 Bait distribution

Aerial baiting was conducted across 12 counties in Pennsylvania with 810,167 baits distributed at a density of 75 baits/km^2^ as part of a larger ORV campaign. In Mercer and Crawford counties where our study was conducted, 224,760 baits were distributed (Fig. 1). Actual bait densities for the 75, 150, and 300 baits/km^2^ treatment areas were 54, 122, and 210 baits/km^2^, respectively.

### 3.2 Trapping

Pre-ORV trapping resulted in a total of 139 raccoons, with slightly more males (n = 76) than females (n = 63) captured (Table 2). Based on our aging criteria, 87 raccoons were classified as adults, seven as juveniles and 45 as unknown.

**Table 2:** The number of raccoons (*Procyon lotor*) collected in Mercer and Crawford counties, Pennsylvania in 2002 at three study areas of varying oral rabies vaccination bait density prior to and after baiting. The rabies virus neutralizing antibodies based on the rapid fluorescent foci inhibition test with 95% confidence intervals (CI), are included as well as the percentage tetracycline biomarker detected.

Bait density (baits/km^2^)	Study area	Pre-bait		Post-bait	
		n, % seropositive (95% CI)	n, % biomarker positive	n, % seropositive (95% CI)	n, % biomarker positive
75	3–1	15, 20.0 (7.1–45.2)	4, 0	12, 16.7 (4.7–44.8)	12, a16.7
	3–2	14, 7.1 (1.3–31.5)	7, 0	13, 15.4 (4.3–42.2)	12, 41.7
	3–3	17, 11.8 (3.3–34.3)	4, 0	20, 0 (0–19.2)	19, 21.1
	Sub-total	46, 13.0 (6.2–25.7)	15, 0	45, 8.9 (3.5–20.7)	43, 25.6
150	2–1	34, 5.9 (1.6–19.1)	17, 5.9	37, 8.1 (2.8–21.3)	36, 33.3
	2–2	9, 11.1 (2.0–43.5)	5, 0	12, 16.7 (4.7–44.8)	12, 50.0
	2–3	19, 5.3 (0.9–24.6)	4, 0	12, 33.3 (13.8–60.9)	12, 58.3
	Sub-total	62, 6.5 (2.5–15.5)	27, 3.7	61, 14.8 (8.0–25.7)	61, 41.0
300	1–1	8, 0 (0–32.4)	5, 20.0	15, 20.0 (7.1–45.2)	15, 40.0
	1–2	7, 0 (0–35.4)	2, 0	21, 9.5 (2.7–28.9)	20, 40.0
	1–3	16, 0 (0–19.4)	8, 0	13, 30.8 (12.7–57.6)	13, 46.2
	Sub-total	31, 0 (0–11.0)	15, 6.7	49, 18.4 (10.0–31.4)	48, 41.7

a Not the same two post-bait seropositive raccoons.

Post-ORV trapping yielded 150 newly captured raccoons and five recaptures (Table 2). The number of males (n = 77) trapped was almost equal to the number of females (n = 76; 2 unknown). Of 155 raccoons, 55 were identified as adults and 57 as juveniles with 43 of unknown age. The five recaptured raccoons were located in the same cells during post-bait trapping as they were during pre-bait trapping.

Non-target species captured included 209 opossums (*Didelphis virginiana*), 89 woodchucks (*Marmota monax*), 45 striped skunks (*Mephitis mephitis*), 31 cats (*Felis catus*), 20 eastern cottontail rabbits (*Sylvilagus floridanus*), 10 squirrels (species not reported), one dog (*Canis lupus*), one red fox (*Vulpes vulpes*), one common grackle (*Quiscalus quiscula*), one Norway rat (*Rattus norvegicus*), and one least weasel (*Mustela nivalis*).

### 3.3 Rabies diagnostics

**Fig. 3:**
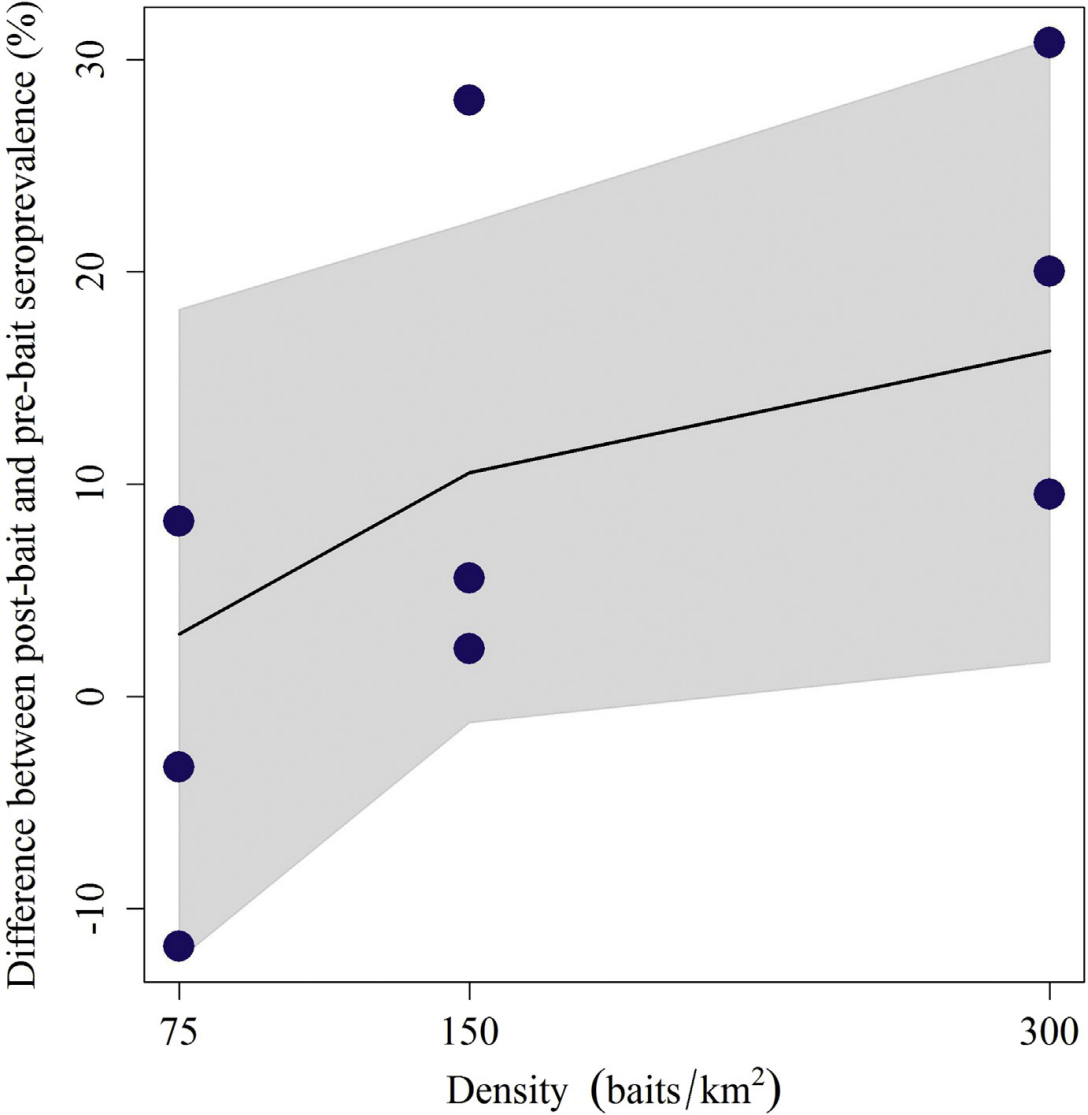
The difference observed between pre-bait and post-bait rabies virus neutralizing antibody (RVNA) seroprevalence in raccoons captured in each of three study zones where oral rabies vaccine bait densities of 75, 150 and 300 baits/km^2^ in Pennsylvania in 2002. Each dot represents one replicate site, with three sites per treatment zone. The model averaged estimate of change in RVNA seroprevalence as a function of bait density is shown (solid line) with 95% confidence intervals (shaded region).

Post-bait RVNA seroprevalence was highest in the study area baited at 300 baits/km^2^ (18.4%; 95% confidence interval (CI) 10.0–31.4), followed by 150 baits/km^2^ (14.8; 95% CI: 8.0–25.7; Table 2). The top model for explaining the change in raccoon RVNA seroprevalence was a linear effect of bait density, and indicated that distribution of higher bait density resulted in higher seroconversion. The only competitive models (within 2 ΔAICc) were the model with the indicator of 75 baits/km^2^ and the null model. The model results suggest that the linear bait density effect model was 50% more likely than either the 75 baits/km^2^ or the null model (0.30, 0.22 and 0.21 AICc weights, respectively, Table 3) to be the true model within our candidate set. The model averaged estimates show that at targeted bait densities of 150 or 300 baits/km^2^, the difference in antibody prevalence is greater than when the target bait density is 75 baits/km^2^ (Fig. 3). The results also suggest that the difference in antibody prevalence for 300 baits/km^2^ is slightly greater than 150 baits/km^2^, but the difference is not as pronounced as that between 75 and 150 baits/km^2^ (Table 3; Fig. 3). We reran the linear bait density effect model with the actual bait densities and confirmed that the results and interpretation did not change.

**Table 3:** Model selection results for the difference in rabies virus neutralizing antibody prevalence of raccoons (*Procyon lotor*) captured in Pennsylvania prior to and after oral rabies vaccine baiting in 2002. Models are compared using the second-order Akaike's Information Criteria (AICc) with more support for smaller values.

Model	K^a^	AICc	ΔAICc^b^	ω^c^	-2LL^d^
Density^e^	3	−5.49	0	0.30	8.14
Den75^f^	3	−4.88	0.60	0.22	7.84
Null	2	−4.77	0.72	0.21	5.38
Den300^g^	3	−3.08	2.40	0.09	6.94
Age	3	−1.03	4.46	0.03	5.91
Sex	3	−0.70	4.79	0.03	5.75
Sex + Density	4	−0.61	4.88	0.03	9.31
Weight	3	−0.01	5.48	0.02	5.40
Sex + Den75	4	0.09	5.58	0.02	8.95
Denfac^h^	4	1.29	6.77	0.01	8.36

a Number of parameters.

b Difference in AICc from the top model.

c AICc model weight.

d Log-likelihood.

e Linear relationship of density.

f Indicator of 1 if density was 75, otherwise it was 0.

g Indicator of 1 if density was 300, otherwise it was 0.

h Density as a categorical effect.

Females and males were equally likely to demonstrate a RVNA response, as were adults and juveniles. Among the five recaptured raccoons, none were RVNA seropositive during pre-bait sampling, but two were RVNA seropositive during post-bait sampling. All but one of the 154 heads submitted for testing were rabies negative. The single rabid animal displayed agitated and aggressive behaviors at the time of capture (Fig. 1).

### 3.4 Tetracycline analysis

Only the null model was competitive for tetracycline response (Table 4). The impacts of age, sex, weight, and bait density were not more parsimonious models for explaining the variation in tetracycline than the intercept only model. A biomarker was more likely to be detected in raccoons captured in the 150 or 300 baits/km^2^ density areas compared to 75 baits/km^2^ (Table 2).

**Table 4:** Model selection results for the difference in tetracycline rate of raccoons (*Procyon lotor*) captured in Pennsylvania prior to and after oral rabies vaccine baiting in 2002. Models are compared using the second order Akaike's Information Criteria (AICc) with more support for smaller values.

Model	K^a^	AICc	ΔAICc^b^	ω^c^	-2LL^d^
Null	2	−3.81	0	0.47	4.91
Sex	3	−1.54	2.27	0.15	6.17
Den75^e^	3	−1.21	2.60	0.13	6.01
Weight	3	0.09	3.90	0.07	5.35
Density^f^	3	0.74	4.55	0.05	5.03
Age	3	0.97	4.78	0.04	4.91
Den300^g^	3	0.99	4.80	0.04	4.91
Sex + Den75	4	3.39	7.21	0.01	7.30
Denfac^h^	4	4.99	8.80	0.01	6.51

a Number of parameters in the model.

b Difference in AICc from the top model.

c AICc model weight.

d Log-likelihood.

e Indicator of 1 if density was 75, otherwise it was 0.

f Linear relationship of density.

g Indicator of 1 if density was 300, otherwise it was 0.

h Density as a categorical effect.

### 3.5 Raccoon density estimate

Raccoon densities were estimated for each zone with 6.0 raccoons/km^2^ (95% confidence interval (CI): 3.1–11.9) in zone 1, 9.6 raccoons/km^2^ (95% CI: 5.0–18.4) in zone 2, and 7.7 raccoons/km^2^ (95% CI: 4.0–14.9) in zone 3. There were large uncertainties in the estimates due to the low recapture rates (0.06, 95% CI 0.03–0.09) compared to initial capture rates (0.44, 95% CI 0.33, 0.55).

## 4 Discussion

The RVNA response increased with increasing bait density (Fig. 3) despite the relatively small number of raccoons captured at each study site (Table 2). While there was clearly site variation among replicates, the trend suggests that increasing bait density will result in increasing raccoon population RVNA seroprevalence. However, since cost is an important consideration when designing an ORV program, the increase in RVNA seroconversion must be sufficient to justify the added expense.

Actual bait densities were lower than the targets for all treatment areas (54, 122, and 210 baits/km^2^). Deviation from target densities is expected since baiting is restricted over selected landscape features such as buildings, residential areas, pastures with livestock, pools and large bodies of water. Despite fewer baits distributed than planned, the treatment effect between 75–150 baits/km^2^ was likely unaffected because there was a still a two-fold increase in the number of baits.

In a similar study conducted in northeastern Ohio, a minimal increase in antibody prevalence was observed between 75–150 baits/km^2^ [34], which was different than the more pronounced effect we observed between 75 and 150 baits/km^2^. However, this apparent difference between the results of our respective studies was more likely attributed to the minimal difference between the actual bait densities that were achieved in Ohio (89 and 131 baits/km^2^) [34] versus the greater than two-fold increase in our study (54 and 122 baits/km^2^). Conversely, in the same Ohio study, seroprevalence was significantly greater when 300 baits/km^2^ was compared to 150 baits/km^2^, whereas the effect we observed between these two bait densities appeared to be minimal (Fig. 3). This was likely attributed to the difference in actual baits distributed in Ohio (131 and 270 baits/km^2^) versus our study (122 and 210 baits/km^2^), when target densities of 150 and 300 baits/km^2^ were applied. Regardless, distributing 300 baits/km^2^ would likely be cost prohibitive and best reserved for special circumstances requiring contingency actions, such as in epizootic areas or a breach of the currently defined ORV zone [34, 35].

Though we observed a slight advantage to baiting at the target density of 150 baits/km^2^, RVNA seroprevalence remained well below the levels indicated to interrupt RABV transmission [36]. To prevent RABV spread, there is a threshold value of immunized animals that need to be vaccinated per unit area [37]. A vaccination immunity level of >60% has been estimated as necessary for prevention and control [18], which is much higher than the apparent immunity observed in any of our study areas. Since raccoons are not territorial and typically survive two to three years [38], the number of baits distributed in each treatment area would have been expected to be sufficient to induce immunity in the calculated densities of raccoons detected in each zone. However, since we only examined the study areas for one year, we are unsure whether we would have observed a cumulative effect of baiting that has been observed in other multi-year studies [34], and whether it would have resulted in a level closer to those reported as necessary to interrupt rabies transmission. In fact, in some cases RVNA seroprevalence was actually lower after baiting (Table 2). Baits were distributed from August 16–18, and then post-bait trapping was conducted approximately four weeks later (beginning September 16^th^). Given this interval, there was sufficient time for biomarker deposition, but some serum samples may have been collected prior to the peak induction of detectable antibodies [39]. The titers identified in the RVNA positive raccoons were highly variable and ranged from 6 to >56. While there is no minimum titer that is indicative of protection against rabies virus, it is unclear whether all animals with RVNA titers were vaccinated or if the detection was an immunological response to a RABV exposure [40, 41, 42]. Although our study was conducted in ORV naïve areas, RVNA antibody was still detected in most of the study areas prior to baiting (Table 2). This was probably attributed to circulation of raccoon RABV in the area which is almost always fatal to raccoons [43], but likely provided immunity to a few animals that recovered from infection. In the two years prior to our study, terrestrial rabies cases were reported in Mercer and Crawford counties in felines and raccoons (http://www.agriculture.pa.gov/Protect/AHDServices/Pages/Rabies.aspx).

Cost effective ORV programs result in 70% of the individuals in the target population consuming at least one bait [44], with the goal of oral contact with the vaccine and subsequent production of RVNA; however, in our study, RVNA were detected in <35% of raccoons captured post ORV. Our raccoon density estimates (6.0–9.6 raccoons/km^2^) were slightly lower than a study conducted in similar habitats in neighboring counties in Ohio in 1998–1999 where raccoon density estimates ranged from 9.8 to 13.9 raccoons/km^2^ (NRMP, unpublished data). Based on the number and variety of non-target species that we captured, non-target competitors for baits should also be considered when planning ORV campaigns. In our study areas there were nearly as many opossums (n = 209) captured as raccoons (n = 294) and at least 10 opossums were captured in each study site. Though we did not sample non-target species for biomarker or RVNA presence, previous studies have reported bait consumption by non-targets including opossums [45, 46], meaning fewer baits were likely available to raccoons.

Our results suggest much higher bait acceptance than vaccine uptake when comparing the serology and biomarker results (Table 2). A similar result was observed in a study conducted in northern Ohio when premolars were used to determine tetracycline deposition and RVNA was used to assess immune response to vaccination [47]. Due to the structure of the fishmeal polymer ORV bait, raccoons may have consumed the bait containing the biomarker without adequate oral contact with the sachet containing the vaccine. In other words, detection of a biomarker does not necessarily indicate that the raccoon was vaccinated. In addition, tetracycline is a common feed additive and antibiotic used for livestock (i.e., cattle, swine) [48], and has been detected in raccoons and other wildlife at low levels [49]. Tetracycline deposition occurs in areas of new bone growth meaning that fluorescence is most likely to be observed in younger animals and can persist for long periods of time [50]. Despite this, our results indicate that bait acceptance was nearly double the RVNA prevalence at each of our study sites.

Since the density of baits distributed has a direct correlation not only on the cost, but also with the ORV campaign effectiveness, it is essential to understand all of the underlying factors that affect the outcome of bait and vaccine uptake. Components such as the densities of target and non-target species, and the most effective method of bait distribution to reach adequate vaccination coverage of the target species should be considered when developing an ORV strategy. Consideration and incorporation of these variables is necessary to achieve raccoon rabies elimination.

## Declarations

### Author contribution statement

Kerri Pedersen, Amy J. Davis, Amy T. Gilbert: Analyzed and interpreted the data; Wrote the paper.

Brandon S. Schmit: Conceived and designed the experiments; Performed the experiments; Wrote the paper.

Thomas J. DeLiberto, Dennis Slate: Conceived and designed the experiments; Wrote the paper.

Jason R. Suckow, Robert L. Hale: Performed the experiments; Wrote the paper.

Richard B. Chipman: Contributed reagents, materials, analysis tools or data; Wrote the paper.

### Funding statement

This research did not receive any specific grant from funding agencies in the public, commercial, or not-for-profit sectors.

### Competing interest statement

The authors declare no conflict of interest.

### Additional information

No additional information is available for this paper.
